# Human Organoids and Organs‐on‐Chips for Addressing COVID‐19 Challenges

**DOI:** 10.1002/advs.202105187

**Published:** 2022-02-02

**Authors:** Yaqing Wang, Peng Wang, Jianhua Qin

**Affiliations:** ^1^ Division of Biotechnology Dalian Institute of Chemical Physics Chinese Academy of Sciences Dalian 116023 China; ^2^ Beijing Institute For Stem Cell and Regeneration Medicine Beijing 100101 China; ^3^ CAS Center for Excellence in Brain Science and Intelligence Technology Chinese Academy of Sciences Shanghai 200031 China; ^4^ University of Chinese Academy of Sciences Beijing 100049 China

**Keywords:** coronavirus disease 2019, human models in vitro, organoids, organs‐on‐chips, severe acute respiratory syndrome coronavirus 2

## Abstract

Coronavirus disease 2019 (COVID‐19), caused by severe acute respiratory syndrome coronavirus 2 (SARS‐CoV‐2), poses an imminent threat to our lives. Although animal models and monolayer cell cultures are utilized for pathogenesis studies and the development of COVID‐19 therapeutics, models that can more accurately reflect human‐relevant responses to this novel virus are still lacking. Stem cell organoids and bioengineered organs‐on‐chips have emerged as two cutting‐edge technologies used to construct biomimetic in vitro three‐dimensional (3D) tissue or organ models. In this review, the key features of these two model systems that allow them to recapitulate organ physiology and function are introduced. The recent progress of these technologies for virology research is summarized and their utility in meeting the COVID‐19 pandemic is highlighted. Future opportunities and challenges in the development of advanced human organ models and their potential to accelerate translational applications to provide vaccines and therapies for COVID‐19 and other emerging epidemics are also discussed.

## Graphical abstract

Stem cell organoids and organs‐on‐chips are two cutting‐edge technologies that allow to recapitulate human organ physiology and function. This review summarizes the key features and progress of these technologies in addressing the Coronavirus disease 2019 (COVID‐19). The future opportunities and challenges in the development of high fidelity 3D organ model systems for accelerating virology research are also discussed.

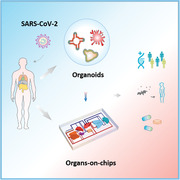

## 1 Introduction

Coronavirus disease 2019 (COVID‐19) has been one of the most serious pandemics in human history. Since its outbreak in December 2019, it has led to considerable economic and social losses worldwide.^[^ ^1^, ^2^, ^3^ ^]^ Severe acute respiratory syndrome coronavirus 2 (SARS‐CoV‐2), which shares extensive homology with SARS‐CoV, is the etiologic agent of COVID‐19. Unlike common respiratory infectious diseases, COVID‐19 is lethal as well as contagious. COVID‐19 patients can be asymptomatic or exhibit a wide spectrum of symptoms, including respiratory distress, coagulopathy, gastrointestinal tract symptoms (abdominal pain or diarrhea), and even systemic injury of multiple organs.^[^ ^1^, ^4^, ^5^ ^]^ SARS‐CoV‐2 has rapidly evolved and multiple mutations have emerged,^[^ ^6^, ^7^, ^8^ ^]^ which posed an increased risk to global health. For example, the SARS‐CoV‐2 Delta variant (B.1.617.2) was identified in late 2020 and spread throughout India.^[^ ^9^, ^10^ ^]^ Compared with the original coronavirus strain, the Delta variant has increased transmissibility and could exhibit higher viral load and immune evasion in COVID‐19 patients.^[^ ^11^ ^]^ Although several types of vaccines against SARS‐CoV‐2 have been developed and are approved for human use, some variants exhibit a reduced sensitivity to these vaccines.^[^ ^12^, ^13^, ^14^, ^15^, ^16^ ^]^ Thus, there is an urgent need to comprehensively understand the pathogenesis of COVID‐19 and identify potential targets for the development of effective therapies.

Animal models and monolayer cell cultures are commonly used in virology studies, and many of these models have been used to investigate SARS‐CoV‐2 infection and screen candidate drugs and vaccines.^[^ ^17^, ^18^, ^19^, ^20^, ^21^ ^]^ Angiotensin‐converting enzyme 2 (ACE2) is considered as the cell‐entry receptor for SARS‐CoV.^[^ ^22^ ^]^ Rodent models, such as transgenic mice that express human ACE2, displayed typical pneumonia histopathology as observed in human with SARS‐CoV‐2 infection, which confirmed the viral pathogenicity.^[^ ^21^ ^]^ However, the mice are not the natural host for this virus, thus they are unable to adequately reflect the human response to SARS‐CoV‐2 infection. Nonhuman primates including rhesus macaques and cynomolgus monkeys have also been instrumental in studying organism responses and testing drugs,^[^ ^23^, ^24^, ^25^ ^]^ but these animals often exhibit symptoms that are substantially different from those of humans. Additionally, monolayer cell cultures, such as Vero E6 and Caco‐2 cell lines, have been widely used for the isolation and replication of SARS‐CoV‐2 and studying the mechanism of viral invasion.^[^ ^26^ ^]^ Previous studies have identified that the host receptor ACE2 and transmembrane serine proteinase 2 (TMPRSS2) are used for cell invasion of SARS‐CoV‐2 via spike protein binding.^[^ ^18^, ^27^, ^28^, ^29^ ^]^ However, the simple cell line cultures often lack cell–cell interactions and exhibit epigenetic and functional differences from native tissues. These limitations highlight the urgent need to establish physiologically relevant models of viral infection for developing effective therapies.

**Figure 1:**
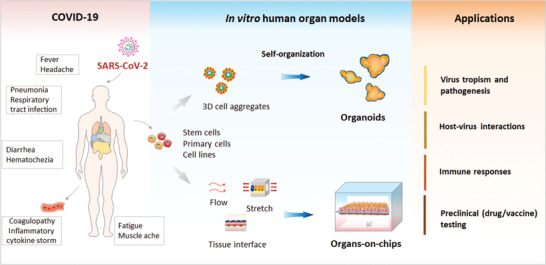
Schematic illustration of in vitro human organ models for COVID‐19 research. COVID‐19, caused by SARS‐CoV‐2 virus, clinically presents a wide spectrum of symptoms, such as fever, pneumonia, abnormal pain, and coaulopathy involving different organs. Organoids are 3D multicellular clusters derived from human stem cells (e.g., ASCs and PSCs) by self‐organization, resembling native tissues. Organ‐on‐a‐chip is a bioengineered microfluidic cell culture device that can mimic cellular microenvironment (e.g., fluid flow, stretch, and tissue interface), recapitulating the functional units of human organs. These two physiologically relevant tissue/organ model systems can be used to study SARS‐CoV‐2 pathogenesis and human‐relevant responses, facilitating their potential applications in disease modeling, drug/vaccine development, immune responses, virus transmission, host‐virus interactions, and personalized therapy.

Significant advances in the development of organoids and organs‐on‐chip technologies have facilitated the construction of in vitro near physiological 3D tissues and organs. Organoids are three‐dimensional (3D) multicellular tissues by the self‐organization of stem cells relying on developmental biology principles.^[^ ^30^, ^31^, ^32^ ^]^ In contrast, organs‐on‐chips are in vitro microfluidic cell culture devices that contain microchannels inhabited by living cells, evolving from microfabrication technologies and bioengineering strategies.^[^ ^33^, ^34^, ^35^ ^]^ They can recreate the miniaturized functional units of various organs, such as the lung, intestine, or neural networks. Organoids and organs‐on‐chips have both shown the ability to reflect the pathophysiology and host responses to distinct diseases, which may provide new opportunities for COVID‐19 research (**Figure** **1**). In this review, we provide a summary on the emerging state‐of‐art organoids and organs‐on‐chips technologies that enable the study of viral infections, and emphasize their progresses in COVID‐19 research. We also discuss the prospects and challenges of building advanced human organ models by integrative engineering strategies to combat the emerging infectious diseases and future epidemics.

## 2 Key Features of Organs‐on‐Chips and Organoids Technologies

The development and regeneration of organs or tissues in vivo are regulated by the spatial cooperation of inherent genetic reprograms of cells and the external niche. Organs‐on‐chips and organoids have emerged as two major technologies to build in vitro 3D tissue or organ models to better mimic human physiology and functions. These two distinct model systems recapitulate the critical tissue‐specific properties at multi‐scales relying on developmental biology and bioengineering principles, respectively, which bridge the gap between animal models and monolayer cultures in existing methods.

### 2.1 Organs‐on‐Chips

Organs‐on‐chips are in vitro microphysiological systems that recreate the functional units of living organs by culturing living cells within a microfluidic device.^[^ ^35^, ^36^ ^]^ Generally, the cell sources in the organ chip models include cell lines, primary cells, and stem cells. By precise control of fluid flow, mechanical cues, and multicellular interactions, organs‐on‐chips can mimic in vivo cellular microenvironments and key functional properties of native organs.^[^ ^33^, ^34^ ^]^ In the past decade, much progress in organs‐on‐chip technology has been made to construct various biomimetic models of human organs, such as the intestine,^[^ ^37^, ^38^ ^]^ heart,^[^ ^39^, ^40^ ^]^ liver,^[^ ^41^, ^42^ ^]^ and lung.^[^ ^35^, ^43^, ^44^ ^]^ These models have considerable potential for disease studies, drug testing, and virology applications.

The key cues provided from the extracellular microenvironment that direct the cellular behaviors and functions of certain tissues or organs include biochemical factors (e.g., cytokines, oxygen, and nutrients), physical factors (e.g., shear stress, mechanical forces, and electrical signals), and cell–cell/cell–matrix interactions. Vasculature perfusion is a crucial aspect in maintaining the morphology and functionality of in vivo tissues and organs, as it enables nutrient exchange and provides shear stress. Perfusion flow in micrometer‐sized channels of the chip device is beneficial to cell survival and increase the physiological relevance of 3D organ cultures in a dynamic microenvironment. The circulation of media through the compartmentalized microfluidic chip enables the interplay between multiple cell types. The chips can also manipulate cell behaviors (e.g., proliferation, migration, and differentiation) by spatiotemporal control over the gradients of biochemical factors or oxygen tension.

In addition to biochemical factors, the investigations of mechanical signals, such as tension deformations and stretch, have broadened our in‐depth comprehension of cell behaviors. Organs‐on‐chip technology can enable cyclic mechanical deformation to be integrated into tissues, such as engineering lung^[^ ^35^ ^]^ and gut on chips,^[^ ^37^ ^]^ which has played a significant role in reflecting human organ physiology and pathology. Other biophysical signals, such as electrical and optical stimuli, can also be introduced into organ chips to direct cell behaviors and monitor output signals. For example, electrical signals and optogenetics have been used to pace cardiac tissue and functionally assess cardiac contraction.^[^ ^45^, ^46^ ^]^ Tissue barriers in vivo play crucial roles in intricate interactions between various tissues and organs in the physiological microenvironment. Organ chips containing compartmentalized chambers sandwiched by porous membranes (e.g., polycarbonates and PDMS) can reproduce tissue–tissue interfaces when distinct cell types are co‐cultured. Recently, diverse organ chips with tissue interfaces have been established to recapitulate functional tissue barriers, such as the lung alveolar air–liquid barrier,^[^ ^35^ ^]^ blood–brain barrier (BBB),^[^ ^47^ ^]^ and placental barrier.^[^ ^48^ ^]^ These models contribute to the exploration of the intricate cross talk between various human cell types and tissues, and the pathophysiological responses to inflammation or infections.

### 2.2 Organoids

Organoids are complex 3D structures derived from pluripotent stem cells (PSCs) or tissue‐resident adult stem cells (ASCs) via self‐organization.^[^ ^30^, ^31^ ^]^ They can mimic the key features of native organs in terms of multicellular compositions, architectures, and functionalities. Recent years have witnessed a revival of organoids that sought to elucidate organogenesis underlying developmental biology. Small intestinal organoids (SIOs) derived from intestinal ASCs were generated by Hans Clevers's group in 2009,^[^ ^49^ ^]^ demonstrating the intrinsic capacity of stem cells to self‐organize into tissue‐specific 3D structures that resemble native organs. Over the past decade, multiple types of organoids have been successfully generated to delineate the physiological hallmarks of developing organs in humans, such as the brain,^[^ ^50^, ^51^ ^]^ intestine,^[^ ^49^, ^52^ ^]^ liver,^[^ ^53^ ^]^ and kidney.^[^ ^54^, ^55^ ^]^ Organoids recapitulate many biological parameters of tissue development, such as the organization of heterogeneous cells and cell–cell/cell–matrix interactions. Compared with 2D cultures and in vivo models, organoids are more amenable to the manipulation of stem cell niche components and genome editing. The versatile nature and potential of organoid models facilitate a range of biomedical applications, including investigations of tissue renewal, organ development, disease etiology, viral infection, and drug discovery.

Despite the potential of organoid models in biomedicine, they often represent single or partial properties of native tissues, some major limitations remain. Generally, the process of organoids formation is spontaneous relying on the self‐organization of stem cells in a 3D extracellular matrix. The animal‐derived matrices (e.g., Matrigel) have been widely used as scaffolds for culturing organoids and to promote their further differentiation. However, the batch‐to‐batch variations and ill‐defined protein compositions of these matrices can lead to the generation of organoids with random configurations, high variability, and low reproducibility. Moreover, most organoid models lack blood vessel networks and immune system, thus they still cannot fully mirror the authentic architecture and functionality of in vivo human organs. These limitations may impede the production of organoids in a controlled microenvironment and their use as faithful models for studying human organ physiopathology in vitro.

As the fields of bioengineering, materials and cell biology have progressed, various approaches have been used to engineer organoids by steering stem cell behaviors and control of cellular microenvironment. Organoids‐on‐a‐chip is emerging as a nascent technology by combining organoids and organ on chips to construct 3D models with higher fidelity.^[^ ^56^, ^57^, ^58^, ^59^ ^]^ Given the limitations of existing organoids system, organ chips technology could provide a strong functionality to control dynamic niche and guide stem cell differentiation into tissue‐specific organoids by spatiotemporally controlling biochemical and physical cues. A micropillar array chip from Zhu et al. provided an early demonstration of controlled formation of embryoid bodies (EBs) from human induced pluripotent stem cells (hiPSCs) and in situ differentiation into brain organoids on a single device by microfabrication, which may reduce the variability of organoids and manipulate organoids in a high‐throughput manner.^[^ ^56^ ^]^ Recent studies have demonstrated the proof of concept of this technology to engineer functional organoids in a controllable physiological relevant microenvrionment. The mechanical flow was demonstrated to enhance the organogenesis and maturation of PSC‐derived brain, intestine, and islet organoids.^[^ ^60^, ^61^, ^62^ ^]^ The favorable influence of physiological flow on the vasculogenesis was displayed in kidney organoids on chip.^[^ ^63^ ^]^ Other engineering strategies such as biomaterials and biofabrication technologies can also be integrated with organoid and organ chip models to improve their functionality to advance their translational applications.^[^ ^64^, ^65^, ^66^, ^67^ ^]^ The use of defined hydrogels served as could mimic native 3D matrices and steer organoid morphogenesis by spatiotemporal control over tissue self‐organization and microenvironmental cues.^[^ ^66^, ^68^, ^69^ ^]^ More recently, Gjorevski et al. developed hydrogel microfabrication‐based approaches to spatiotemporally control the morphogenesis of intestinal organoids with defined shape and structure, which may contribute to identify the potential mechanisms of tissue morphogenesis.^[^ ^70^ ^]^ In addition, 3D bioprinting has been a promising biofabrication strategy to produce macro‐scale organoids within biomaterial scaffolds. The centimeter‐scale tissues, including intestinal tube and branched vasculature were recently printed by building tissue blocks and control of tissue assembly.^[^ ^71^ ^]^ But, bioprinting technologies remain challenges in faithfully supporting organoids biology in terms of tissue microarchitecture, cell‐type diversity, and functionality due to the complex organoid self‐organization process. Nevertheless, these strategies hold great potential in substantial improvement in organoids and organ chips models in a controllable manner, which offer possibilities for reproducing in vivo‐like complex tissues or organs.

## 3 Human Organoids and Organs‐on‐Chips for Studying Viral Infection

Organoids and organs‐on‐chips can recapitulate the complex architectures and functionalities of human organs in vitro. Although they are different in terms of cell source, architectural variability, functional features, and scales, both of them are highly useful for studying viral infectious diseases.

Multicellular organoids have been explored for studying tissue tropism to viruses and host pathological responses.^[^ ^51^, ^72^, ^73^, ^74^ ^]^ For example, Zika virus (ZIKV) infections, which became prominent in 2015, can result in severe congenital abnormalities. Previous studies demonstrated the replication of ZIKV in the neural precursors using human cerebral organoids, leading to stunted cortical expansion and microcephaly.^[^ ^51^ ^]^ Influenza viruses, such as the highly human‐infective pandemic 2009 H1N1 virus, can rapidly spread in human populations. Human lung organoids that accommodate four types of airway epithelial cells have been established to evaluate the infectivity of different influenza virus strains.^[^ ^72^ ^]^ In addition, liver organoids derived from human PSCs (hPSCs) have been applied to recapitulate hepatitis B virus (HBV)–host interactions, which may provide a promising individualized infection model to treat individual hepatitis patients.^[^ ^73^ ^]^ These organoid models provide biologically relevant and available platforms for studying the physiology and pathology of human organs and tissues against infection with distinct types of viruses.

Organs‐on‐chips also allow the study of host–pathogen interactions and immune responses in a biomimetic microenvironment.^[^ ^75^, ^76^, ^77^, ^78^ ^]^ For example, a 3D liver chip incorporating primary human hepatocytes has been used to study HBV infection.^[^ ^75^ ^]^ The system recapitulates the HBV life cycle in hepatocytes after viral infection and immune responses mimic that observed in HBV‐infected patients. In addition, a microvessel‐on‐a‐chip was designed to model Ebola hemorrhagic shock syndrome and test the potency of therapeutic drug candidates in a high‐throughput manner.^[^ ^76^ ^]^ The engineered microvessel with Ebola virus infection exhibited disease phenotypes (i.e., albumin leakage) involving signaling pathway alterations. Organs‐on‐chips can reproduce functional tissue barriers owing to their unique ability to form tissue–tissue interfaces. A distal tubule‐on‐a‐chip was built to model the kidney tubular barrier and the pseudorabies virus (PrV) induced kidney disease.^[^ ^77^ ^]^ After PrV infection, this model displayed disordered sodium transporters, a broken reabsorption barrier, and altered microvilli structure, which provide a deep insight into the pathogenesis of virus induced renal dysfunctions.

In general, organoids and organs‐on‐chips offered unique possibilities to recapitulate human relevant responses to viral infections, to explore virus pathogenesis and the development of potential therapies. These organ models could be utilized to obtain a more precise and visual prediction of human pathophysiology in comparison to animal models and traditional cell cultures, thus providing valuable preclinical platforms for viral infection research.

## 4 Progress of Organoids and Organs‐on‐Chips in COVID‐19 Research

During the on‐going COVID‐19 pandemic, organoids and organs‐on‐chips technologies have exhibited their respective utilities in studying SARS‐CoV‐2 infection. In the following section, we outline recent progress in the development of these two models to reflect human organ‐specific responses to SARS‐CoV‐2 infection. We also describe their use in the study of infection kinetics, virus–host interactions, immune responses, and drug assessment in coping with the challenges of COVID‐19.

### 4.1 Studying Virus Tropism in Stem Cell Organoids

#### 4.1.1 Lung Organoids

**Figure 2:**
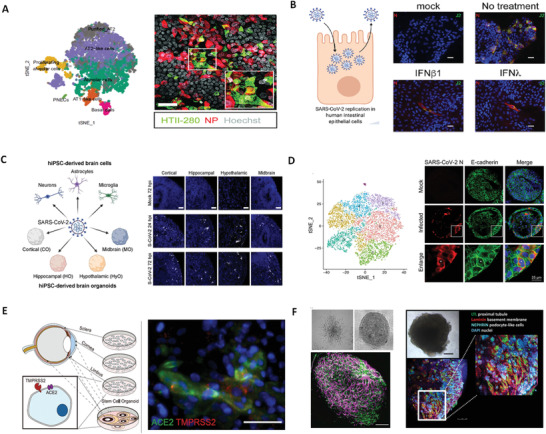
Organoids for modeling SARS‐CoV‐2 infection. A) The alveolar type II cells (HTII‐280) were permissive to SARS‐CoV‐2 infection in the differentiated human lung bud tip organoids at air–liquid interface. Reproduced with permission.^[^ ^83^ ^]^ Copyright 2021, EMBO Press. B) Human colon organoids showed that SARS‐CoV‐2 can infect intestinal epithelium and the infection can be controlled by type I and III interferons. Reproduced with permission.^[^ ^98^ ^]^ Copyright 2020, Cell Press. C) The region‐specific brain organoids derived from hPSCs reveal that choroid plexus epithelial cells were susceptible to SARS‐CoV‐2 infection. Reproduced with permission.^[^ ^104^ ^]^ Copyright 2020, Cell Press. D) Human liver ductal organoids revealed SARS‐CoV‐2 infection in cholangiocytes and virus‐induced cholangiocyte damage. Reproduced with permission.^[^ ^116^ ^]^ Copyright 2020, Springer‐Verlag GmbH and Co. KG. E) A whole‐eye organoid from human embryonic stem cells (hESCs) can be directly infected by SARS‐CoV‐2, mostly notably in limbus. Reproduced with permission.^[^ ^118^ ^]^ Copyright 2021, Cell Press. F) Human capillary and kidney organoids were readily infected by SARS‐CoV‐2, which can be inhibited by human recombinant soluble ACE2 (hrsACE2). Reproduced with permission.^[^ ^115^ ^]^ Copyright 2020, Cell Press.

The lung is the primary target organ for the infection and replication of SARS‐CoV‐2, which is primarily transmitted through the respiratory route. From the upper airways, the virus can enter the lungs and cause respiratory failure and distal alveolar damage in severe COVID‐19 cases. Previous studies have demonstrated the expression of ACE2 and TMPRSS2 that is essential for SARS‐CoV‐2 entry within airway and alveoli epithelium by single‐cell RNA‐sequencing or gene profiles analysis.^[^ ^29^, ^72^, ^79^, ^80^ ^]^ Human lung organoids derived from PSCs or ASCs are effective tools that can recapitulate the respiratory tract histology and functions, which could be used as organotypic models to study SARS‐CoV‐2 kinetics, tropism, and host responses. Recent studies have developed airway and alveoli organoids for investigating SARS‐CoV‐2 infection.^[^ ^81^, ^82^, ^83^, ^84^ ^]^ For example, Chen and colleagues using hPSC‐derived lung organoids revealed that alveolar type II‐like (AT2) cells were permissive to SARS‐CoV‐2 infection and virus‐induced chemokines and cytokines production, which was in accordance with clinical findings involving immune response in COVID‐19 patients.^[^ ^81^ ^]^ Similarly, a human alveolar model and primary lung stem cell‐based alveolospheres both verified the infectability of AT2 cells and induction of interferon responses mediated by SARS‐CoV‐2 (**Figure** **2**A)^[^ ^83^, ^85^ ^]^ Of note, apical ACE2‐containing cells in many AT2 organoids or alveolospheres are located in the interior side. Recently, apical‐out AT2 organoids were established to study SARS‐CoV‐2 infection, facilitating the access of virus to ACE2‐expressing apical cells on the external organoid surface.^[^ ^82^ ^]^

In addition, airway organoids have also been used to study respiratory viruses including SARS‐CoV‐2. Generally, primary airway epithelial cells are cultured at air–liquid interface (ALI) on transwell inserts,^[^ ^86^ ^]^ which enables the differentiation of multiply cell types including ciliated cells, goblet cells, club cells, and basal cells. Recent studies identified ciliated cells as major SARS‐CoV‐2 target cells in small airway organoids at 2D ALI.^[^ ^83^ ^]^ In contrast, a distal human lung organoids model in 3D cultures containing AT2 and basal stem cells revealed club cells as a target population, suggesting that different culture conditions may affect viral tropism.^[^ ^82^ ^]^ Recent findings revealed a gradient of SARS‐CoV‐2 infection in proximal and distal respiratory tract epithelium and a decreased ACE2 expression in alveolar cells,^[^ ^87^ ^]^ indicating that alveolar cells may not be the first infected cell types. Similarly, ASC‐derived lung organoids contained mixed proximodistal epithelia confirmed that airway epithelium was critical for viral infectivity, whereas distal alveolar cells were important for simulating host response in severe COVID‐19.^[^ ^84^ ^]^ These studies revealed that airway epithelium may be infected with SARS‐CoV‐2 prior to the alveoli, with increased ACE2 expression mediated by interferon.^[^ ^80^ ^]^ The lung organoids with mixed cell types provided more views and information about viral infection compared to traditional cell cultures and animal models.

In general, human pulmonary organoids provide a useful model system for understanding SARS‐CoV‐2 pathogenesis and other pathogens targeted the lungs, as well as developing effective therapies. These lung organoids could also be co‐cultured with immune cells or vascular cells, which may enable the study of host‐immune responses to SARS‐CoV‐2 and screen of immunomodulatory drugs. Despite the progress of lung organoid models in SARS‐CoV‐2 research, they still have some limitations. Normally, ACE2 receptors are expressed on the apical side of lung organoids, but 3D organoid cultures usually do not allow virus access to the apical surface. Although some airway ALI cultures may address this issue, they often lack mechanically active physiological microenvironment. Moreover, lung organoids lack typical epithelium‐endothelium tissue interfaces as like in vivo, which cannot reflect the near physiological cross‐talk among distinct cell types after viral infection.

#### 4.1.2 Intestine Organoids

Gastrointestinal symptoms in some COVID‐19 patients^[^ ^88^, ^89^ ^]^ and frequent detection of SARS‐CoV‐2 RNA in anal swabs and stool samples^[^ ^90^, ^91^ ^]^ suggest that the intestine may be another target organ of SARS‐CoV‐2 besides the lungs. Previous studies have shown the high levels of the SARS‐CoV‐2 receptor proteins (ACE2 and TMPRSS2) expression in intestinal cells.^[^ ^80^, ^92^ ^]^ Intestinal organoids have become useful tools for studying enteric infections,^[^ ^93^, ^94^ ^]^ which offer an alternative in vitro model to examine whether SARS‐CoV‐2 could replicate in the human intestine. Several studies have established human ASC‐derived SIO and PSC‐derived colonic organoid models for SARS‐CoV‐2 infection.^[^ ^81^, ^95^, ^96^ ^]^ These models identified that the human intestinal epithelium (enterocytes) can be readily infected with SARS‐CoV‐2, suggesting the intestine as a potential site of SARS‐CoV‐2 replication. Moreover, TMPRSS2 and transmembrane serine proteinase 4 (TMPRSS4) TMPRSS4 were found to promote viral entry into host cells by cleaving the viral spike protein.^[^ ^97^ ^]^ RNA sequencing analysis revealed upregulation of cytokines and chemokines signaling in SARS‐CoV‐2 infected colonic organoids, which may contribute to the disease progression. In infected human SIOs, SARS‐CoV‐2 elicited cytokines upregulation and strong interferon responses. Similarly, Stanifer et al. using primary non‐transformed colon organoids demonstrated that SARS‐CoV‐2 infection can induce interferon‐mediated intrinsic immune responses, and these responses play a crucial role in regulating SARS‐CoV‐2 replication in intestinal epithelium (Figure 2B).^[^ ^98^ ^]^ These findings provide insights into deep understanding the intestinal pathologies observed in COVID‐19 patients involving exacerbated cytokine response and increased viremia. Additionally, a bat intestinal organoid model were recently established to demonstrate the susceptibility of SARS‐CoV‐2 infection and robust viral replication in bat intestinal cells,^[^ ^96^ ^]^ which may contribute to probing the potential origin of this novel zoonotic coronavirus and the possible cross‐species transmission.

#### 4.1.3 Brain Organoids

Neurological complications, such as encephalitis, headache, hypogeusia, and neuropsychiatric ailments, have been observed in a significant number of COVID‐19 patients.^[^ ^99^, ^100^ ^]^ While SARS‐CoV‐2 has been detected in the brain or cerebrospinal fluid of some patients,^[^ ^101^, ^102^ ^]^ its neurotropism and ability to impact brain function is not well understood. PSC‐derived brain organoids offer a feasible tool for probing the neurotropism of SARS‐CoV‐2 and potential pathogenesis.^[^ ^102^, ^103^, ^104^, ^105^, ^106^, ^107^ ^]^ The Ming group investigated the susceptibility of hiPSC‐derived region‐specific brain organoids to SARS‐CoV‐2 infection (Figure 2C).^[^ ^104^ ^]^ They found that choroid plexus epithelial cells showed more viral susceptibility than neurons and astrocytes. They further generated hiPSC‐based choroid plexus organoids and showed productive SARS‐CoV‐2 infection in organoids, leading to increased inflammatory response and brain cell dysfunction.^[^ ^104^ ^]^ Similarly, study by Lancaster's group reported that SARS‐CoV‐2 primarily infected choroid plexus cells while not neurons in brain organoids, leading to damage of blood‐cerebrospinal fluid barrier.^[^ ^106^ ^]^ Inconsistently with above conclusion, several groups reported that SARS‐CoV‐2 had a preferred tropism to neurons in brain organoids.^[^ ^107^, ^108^, ^109^ ^]^ It suggests that different brain organoid regions and culture conditions may influence the viral tropism. Additionally, Wang et al. found the ApoE4 (a genetic risk factor for Alzheimer disease) neurons and astrocytes from hiPSCs were more susceptible to SARS‐CoV‐2 infection, suggesting that ApoE4 may play a causal role in COVID‐19 severity.^[^ ^110^ ^]^ It may help us understand the potential effects of risk factors in different patient populations. In these studies, the researchers directly exposed brain organoids to SARS‐CoV‐2 virus, which may not accurately recapitulate the viral infection path or in vivo virus amount due to the lack of vasculature and intact BBB in brain organoids. The concern regarding whether the neurotropic effects of SARS‐CoV‐2 are due to direct SARS‐CoV‐2 infection on brain cells or associated with the virus's systemic effect still needs to be revealed. Nevertheless, these studies provide insight into the pathognomonic symptoms of COVID‐19 and support the use of brain organoids as a platform for investigating SARS‐CoV‐2 cellular susceptibility, disease mechanisms, and treatment strategies.

#### 4.1.4 Other Organoids

SARS‐CoV‐2 can spread from the lungs to other organs, leading to multi‐organ dysfunction and systemic responses in severe patients.^[^ ^1^, ^4^ ^]^ Previous studies have demonstrated ACE2 expression in multiple extrapulmonary tissues (e.g., heart, kidney, and blood vessel).^[^ ^111^, ^112^, ^113^, ^114^ ^]^ Other types of stem cell organoids, such as the kidney, liver, pancreas, vasculature, eye, and nose organoids, have been established to study the SARS‐CoV‐2 tropism, replication, and immune responses in distinct targeted organs.^[^ ^106^, ^115^, ^116^, ^117^, ^118^ ^]^ For example, liver ductal organoids containing ACE2^+^/TMPRSS2^+^ cholangiocytes showed a high susceptibility of SARS‐CoV‐2 infection that induced direct cholangiocyte injury and bile acid accumulation (Figure 2D).^[^ ^116^ ^]^ These observations may be associated with COVID‐19 patient liver damage. In addition, human nose organoid established from nasal wash and turbinate swab samples recapitulated host responses to SARS‐CoV‐2 infection, including ciliary damage, immune responses, and mucus hypersecretion, which could be used as a non‐invasive alternate model to lung organoids.^[^ ^117^ ^]^ Recently, researchers found the expression of SARS‐CoV‐2 antigens in COVID‐19 patient ocular surface tissue.^[^ ^118^ ^]^ They further established the hESC‐derived eye organoid model and identified limbus cells as a target for viral replication in accompany with chemokine production and diminished interferon signaling (Figure 2E).

Collectively, organoids have displayed their practicability in the study of SARS‐CoV‐2 infectivity since the cell‐entry receptor (e.g., ACE2 and TMPRSS2) of the virus are abundant in most of organoids. The summary of various stem cell organoids models used for COVID‐19 research can be seen in **Table** **1**. These organoids can be used as an attractive platform to study virus tropism and host pathological responses to this novel virus, thus may help for understanding potential disease mechanism and speeding up drug development. However, organoid models remain limitations, such as the lack of immune systems. In future, organoids could also be co‐cultured with organ‐specific immune cells, which may enable the study of immunological responses to SARS‐CoV‐2 and explore the activity and effectiveness of immunomodulatory drugs to treat COVID‐19. Moreover, organoids with genomic editing or multi‐omics analysis could further reveal the viral pathogenesis involving altered a series of molecular and cellular events.

**Table 1:** Summary of existing organoid models used for COVID‐19 research

Types	Cell source	SARS‐CoV‐2 tropism	Disease outcomes
Liver^[^ ^106^, ^116^ ^]^	hPSC^[^ ^106^ ^]^ Human primary liver bile duct‐derived progenitor cells^[^ ^116^ ^]^	Human hepatocyte and cholangiocyte organoids^[^ ^106^ ^]^ Human liver ductal organoids^[^ ^116^ ^]^	Induced chemokine responses^[^ ^106^ ^]^ Induced cell death of cholangiocytes^[^ ^116^ ^]^ Impaired barrier and bile acid transporting functions of cholangiocytes^[^ ^116^ ^]^
Kidney^[^ ^115^ ^]^	hiPSCs	Kidney organoids	Active viral replication in kidney organoids^[^ ^115^ ^]^ Reduced SARS‐CoV‐2 infections using soluble human ACE2^[^ ^115^ ^]^
Vessel^[^ ^115^ ^]^	hiPSCs	Capillary organoids	Active viral replication in capillary organoids^[^ ^115^ ^]^ Inhibiting SARS‐CoV‐2 infections using soluble human ACE2^[^ ^115^ ^]^
Lung^[^ ^81^, ^82^, ^83^, ^84^, ^85^ ^]^	hPSC^[^ ^81^ ^]^ hASC^[^ ^82^, ^83^, ^84^, ^85^ ^]^	Alveolar type II‐like pneumocytes^[^ ^81^, ^82^, ^83^, ^85^ ^]^ Airway cells^[^ ^84^ ^]^ Apical‐out mixed distal lung organoids and club cells^[^ ^82^ ^]^	High‐throughput screening FDA‐approved drugs using lung organoids^[^ ^81^ ^]^ Upregulated IFNs, decreased surfactant proteins and apoptosis in infected AT2 cells, and low‐dose IFN pre‐treatment blocks viral replication in alveolospheres^[^ ^85^ ^]^ Induced type I/III interferon response program^[^ ^83^ ^]^ Alveolar cells were important for mounting the overzealous host immune response^[^ ^84^ ^]^
Intestine^[^ ^81^, ^95^, ^96^, ^98^ ^]^	hASC^[^ ^95^, ^96^ ^]^ hPSC^[^ ^81^ ^]^ Human primary non‐transformed colon organoids^[^ ^98^ ^]^	Multiple colonic cell types, especially enterocytes	Strong induction of a generic viral response program^[^ ^95^ ^]^ Active viral replication, induction of type III IFNs and inflammatory mediators in human enteroids^[^ ^96^ ^]^ Inhibiting SARS‐CoV‐2 infection in colonic organoids with FDA‐approved drugs treatment^[^ ^81^ ^]^ Pretreatment with type I and III IFNs controlled viral infection^[^ ^98^ ^]^
Brain^[^ ^104^, ^105^, ^107^, ^109^ ^]^	hiPSC	Human neural progenitor cells, neurospheres, and brain organoids^[^ ^107^ ^]^ Brain choroid plexus organoids, choroid plexus epithelial cells^[^ ^104^, ^105^ ^]^ Neurons in human brain organoids^[^ ^109^ ^]^	Caused cytotoxicity in the infected hNPCs^[^ ^107^ ^]^ Increased cell death and transcriptional upregulation of inflammatory genes in infected choroid plexus organoids^[^ ^104^ ^]^ Aberrant Tau localization and neuronal cell death^[^ ^109^ ^]^ Disrupted the blood‐ cerebrospinal fluid barrier^[^ ^105^ ^]^
Eye^[^ ^118^ ^]^	hESC	hESC‐derived eye organoids	Eye organoids express ACE2 and TMPRSS2, and can be infected by SARS‐CoV‐2^[^ ^118^ ^]^
Nose^[^ ^117^ ^]^	hASC	Human nose organoids	Viral shedding, ciliary damage, and innate immune responses in infected nose organoids^[^ ^117^ ^]^

### 4.2 Recapitulating Pathophysiology and Host‐Immune Responses in Organ Chips

#### 4.2.1 Lung‐on‐a‐Chip

**Figure 3:**
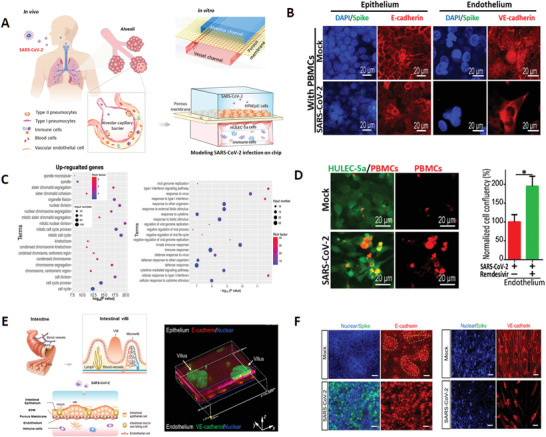
Human lung and intestine chips enable the study of SARS‐CoV‐2 induced tissue injury and immune responses. A) The microengineered alveolus chip consists of an upper alveolar epithelial layer and a lower pulmonary microvascular endothelial layer separated by a porous PDMS membrane. It can mimic the in vivo human alveolar‐capillary barrier by co‐culture of different cell types under fluid flow conditions. Reproduced under the terms of the Creative Commons CC‐BY license.^[^ ^119^ ^]^ Copyright 2020, The Authors. Published by Wiley‐VCH. B) Upon SARS‐CoV‐2 infection on the chip, the epithelium exhibited viral infection and massive replication, but the endothelium did not. C) The transcriptional analysis of host cells after viral infection showed activated innate immune responses in the epithelium and cytokine‐dependent pathways in the endothelium. D) Viral infection caused the recruitment of circulating immune cells and the injury of endothelial cells. E) The biomimetic human gut‐on‐chip was constructed by co‐culture of intestinal epithelial cells, endothelial cells, and immune cells in a multilayered channel under mechanical flow conditions. The intestinal barrier on chip was identified by the intestinal villus‐like structures and the adhesion junction proteins expression in the epithelium and endothelium. Reproduced with permission.^[^ ^126^ ^]^ Cpyright 2020, Science China Press. Published by Elsevier B.V. and Science China Press. F) After SARS‐CoV‐2 infection on the gut chip, the spike protein was expressed primarily in the intestinal epithelium while not in the endothelium, indicating the permissiveness of epithelial cells for viral infection. The intestinal barrier showed obvious morphological changes with injury of intestinal villi and reduced expression of tight junctions (E‐cadherin and VE‐cadherin) in epithelium and endothelium.

To confront the needs of studying viral pathogenesis and the development of effective therapies in COVID‐19 pandemic, several organs‐on‐chips, such as lung chips, have been utilized to recapitulate human‐relevant physiological and pathological responses to SARS‐CoV‐2 infection.^[^ ^119^, ^120^, ^121^ ^]^ A microengineered human lung chip was reported to model the alveolar infection by native SARS‐CoV‐2 and evaluate the efficacy of anti‐viral compound (**Figure** **3**A–D).^[^ ^119^ ^]^ By triculturing alveolar epithelial cells, microvascular endothelial cells and circulating immune cells under fluid flow, this engineered lung chip could reconstitute the key features of human alveolar‐capillary barrier. After inoculating SARS‐CoV‐2 particles in the alveolar channel, the human lung epithelial cells showed higher susceptibility to viral infection than endothelial cells. Clinical trials have suggested that SARS‐CoV‐2 entry into host cells triggers a prodigious immune response and inflammatory cell infiltration in multiple tissues,^[^ ^21^, ^122^, ^123^ ^]^ which often results in cytokine storms. Thus, immune cells may play a crucial role in mediating the systemic inflammatory response, increase damage in the lungs and other organs.^[^ ^124^ ^]^ Our lung infection model demonstrated that SARS‐CoV‐2 triggered the disruption of junction proteins distributions in human epithelial and endothelial cells and increased inflammatory cytokine release in the presence of immune cells, suggesting that immune cells may contribute to the lung barrier injury and exacerbating inflammation. In particular, SARS‐CoV‐2 can induce endothelial impairment in the lung chip contained immune cells, indicating that aggravated immune responses may involve in mediating endothelium injury. These results might explain the pathogenesis of the lung microvascular thrombosis and endotheliitis existed in severe COVID‐19 patients.

Another human lung chip composed of primary alveolar epithelial cells and lung microvascular endothelial cells was also used for SARS‐CoV‐2 infection and identifying the dynamics of virus‐induced vascular damage.^[^ ^120^ ^]^ This study revealed that SARS‐CoV‐2 induced vascular damage occurs even in the absence of immune cells, which was different from our results. It may be partially due to the use of different cell types and experimental conditions. Compared with lung organoids in viral research, the lung chips can recapitulate more complex host responses to SARS‐CoV‐2 infection in human‐relevant manner by integrating with dynamic flow, ALI, and circulating immune cells. Specially, lung chips enable the exposure of virus to alveolar or airway epithelium surface, resembling the respiratory transmission route. Moreover, lung chips enable to reflect the immune responses and cell–cell interactions to viral infection by integrating with immune cells and blood vascular cells, which is often difficult to achieve in existing organoid systems. Yet, the lung chips remain some shortcomings, such as the limited cell types that described in previous paper.^[^ ^119^ ^]^ Nevertheless, the microengineered lung chip model systems could reveal virus‐induced lung injury and study the intrinsic crosstalk between virus and host cells. This may provide useful insights into the dynamics and mechanisms of SARS‐CoV‐2 infection. As COVID‐19 is a systemic disease involving many organs, which may lead to neurological complications in some patients. More recently, a new alveolus‐BBB multi‐organ chip system was proposed to probe the effects of SARS‐CoV‐2 on brain cells following lung infection.^[^ ^125^ ^]^ This work assumed the viral‐induced neuropathological alterations possibly result from systemic inflammation after lung infection, which may provide insights into the mechanisms of COVID‐19.

#### 4.2.2 Intestine‐on‐a‐Chip

The human intestinal barrier contains multiple cell types including intestinal epithelium, vascular cells, and immune cells in a dynamic microenvironment, which can prevent pathogen infection or toxin invasion. To explore the intestinal responses in COVID‐19, a biomimetic human intestine infection chip model was established, which revealed intestinal injury and immune responses induced by SARS‐CoV‐2 (Figure 3E,F).^[^ ^126^ ^]^ The established intestine chip recapitulated the key features of intestinal barrier with villus‐like structure by integrating multicellular components and fluid flow. This model verified that the intestinal epithelium is a potential portal for viral infection and showed viral‐induced barrier damage, including the destructed intestinal villi structure and endothelium junctions. Transcriptome analysis revealed activated immune responses and upregulated inflammatory cytokine‐related genes in epithelium and endothelium. We speculated that the inflammatory factors or paracrine signals released by adjacent infected epithelium may affect the injury of vascular endothelium. These results may provide insights into understanding the gastrointestinal symptoms in COVID‐19 patients.^[^ ^127^, ^128^, ^129^ ^]^ This work still has some limitations, for example, the use of tumor‐derived intestinal epithelial cell line (Caco‐2). The primary intestinal epithelial cells or organoid‐derived cells may be an alternative to reflect the nearly physiology of human intestine in future studies.

Intestinal organoids have showed the susceptibility of SARS‐CoV‐2 in intestinal epithelium,^[^ ^81^, ^95^, ^96^ ^]^ but they often lack physiological relevant microenvironment and intestinal barrier architecture. Previous studies have demonstrated the beneficial effects of fluid flow or mechanical cues on cell differentiation, function, and villi structure formation that are crucial for recapitulating intestinal pathophysiology.^[^ ^38^, ^130^ ^]^ A human intestinal chip showed increased ACE2 protein levels under flow and mechanical deformation conditions, enabling to model enteric coronavirus infection.^[^ ^131^ ^]^ These intestinal chips integrated with immune cells reflected viral‐induced barrier dysfunction and inflammatory response that other in vitro models cannot achieved. They offered useful preclinical platforms for studying virus related pathology and potential therapeutics testing. In addition, previous studies have revealed the potential antiviral capacity and therapeutic value of the gut microbiota in SARS‐CoV‐2 infection.^[^ ^132^ ^]^ It might be helpful to include gut microbiota in intestinal chips to study their roles in COVID‐19 progression. As the potential extra‐respiratory transmission routes remain uncertain, the proposed human intestine model would be useful for investigating viral transmission related to the intestinal tract.

### 4.3 Drug Assessment and Screening

The development and testing of potentially effective drugs for treating COVID‐19 is highly desirable. Currently, drug testing relies heavily on animal models and monolayer cell cultures, but they often inaccurately predict the human responses to drugs. Organoid and organs‐on‐chip platforms as potential disease models can reflect host cell responses to the virus, and they may prove useful for rapidly screening new drugs or identifying potential therapies for COVID‐19.

Infected lung and colonic organoids derived from hPSCs have been used for high‐throughput screening of US Food and Drug Administration (FDA)‐approved drugs (e.g., imatinib, mycophenolic acid), which showed that these drugs can significantly inhibit SARS‐CoV‐2 infection of these organoids.^[^ ^81^ ^]^ Previous studies revealed the induction of type I/III interferons in SARS‐CoV‐2 infected AT2 cells.^[^ ^83^, ^133^ ^]^ Pre‐treatment with a low dose of exogenous interferon could inhibit SARS‐CoV‐2 replication in alveolospheres and bronchioalveolar model. In addition, Monteil et al. used human kidney organoids and blood vessel organoids to identify the effectiveness of soluble human ACE2 (hrsACE2) in inhibiting SARS‐CoV‐2 replication in a dose‐dependent manner (Figure 2F),^[^ ^115^ ^]^ which suggests that hrsACE2 might block the virus from entering target cells. A second‐generation antiandrogen agent, enzalutamide was demonstrated to reduce the expression of TMPRSS2 that mediated SARS‐CoV‐2‐driven entry in prostate cancer cells.^[^ ^134^ ^]^ However, studies on both human lung organoids and lung cancer cells showed that enzalutamide failed to prevent SARS‐CoV‐2 infection due to the androgen receptor‐independent TMPRSS2 expression in lung epithelium. Consistently, in vivo mouse models also demonstrated the lack of antiviral activity of enzalutamide in the lungs.^[^ ^135^ ^]^ These findings revealed that human organoids may be served as an alternative model for animals to evaluate therapeutic efficacy in COVID‐19.

As organs‐on‐chips can recapitulate host responses to virus infection by integrating immune cells and multicellular interactions in a biomimetic microenvironment, they could enable a more accurate preclinical evaluation of new therapeutics and candidate antiviral drugs, as well as drugs repurposing by targeting the immune responses to viral infection rather than the virus itself. Human lung chips have been used to mimic virus infection and identify the clinical efficacy of potential therapeutic drugs. The approved drug remdesivir was assessed in the SARS‐CoV‐2 infected lung chip model, which displayed its ability to inhibit viral replication and alleviate alveolar barrier disruption.^[^ ^119^ ^]^ Clinical trial results showed remdesivir may shorten the recovery time for some COVID‐19 patients, but not significantly reduce mortality. This highlights the urgency of testing and developing other candidates, such as anti‐inflammatory cytokine inhibitors. Another alveolar chip with SARS‐CoV‐2 infection showed that the administration of tocilizumab slowed the loss of barrier integrity by reducing the inflammatory response rather than inhibiting viral replication.^[^ ^136^ ^]^ In addition, a bronchial airway‐on‐a‐chip infected with SARS‐CoV‐2 pseudoparticles identified existing approved drugs (e.g., amodiaquine and toremiphene) as potential viral entry inhibitors. Similarly, amodiaquine showed prophylactic and therapeutic activities in animal models.^[^ ^78^ ^]^ The results suggest that human organ chips may be considered as viable alternatives to animal models in a more physiological relevant manner to identify potential therapeutic drugs. These examples highlight the value of organoid and organs‐on‐chips for expediting the development of new therapeutics and drug repurposing in COVID‐19 pandemic crises. In short, different models have their merits for COVID‐19 research, but many challenges remain. Some of the limitations and advantages of available models for COVID‐19 research are summarized in **Table** **2**.

**Table 2:** The comparisons of different model systems for studying SARS‐CoV‐2 infection

Models	Advantages	Limitations
2D cell cultures (e.g., cell lines, primary cells, and tissue explants)	Often be highly susceptible to viruses Convenient for the isolation and replication of viral particles including SARS‐CoV‐2 Studying mechanism of viral invasion Large‐scale screening anti‐viral drugs	Lacking cell–cell/matrix interactions and complex 3D tissue organization Different from native tissues in terms of gene profiles, epigenetics, and functions Limited sources and short viability of primary cells/tissues Difficult to study virus tropism
Organoids	Recapitulating key features of organ development Modeling viral life cycle Amenable to extended cultivation and manipulation Long‐term preservation of cell phenotype and genotype in vitro Available for studying virus tropism with multiple cell types High‐throughput drug screening	Uncontrolled biochemical and biophysical environmental cues High variability Lack of relevant mechanical signals, such as blood perfusion and air flow Often use of ill‐defined animal‐derived matrices (e.g., Matrigel) Lack of tissue–tissue interfaces Lack of immune cells or vascular structure Difficult access of virus to apical epithelium surface
Organs‐on‐chips	Mimicking in vivo‐like tissue microenvironment Recapitulating the human‐relevant tissues or organs physiology and pathology Precise control of mechanical cues (e.g., fluid flow) Mimicking tissue–tissue interfaces Studying cell–cell interactions by cell co‐cultures Enable apical surface accessibility of virus Studying host‐immune responses to viral infection and viral evolution In situ and real‐time imaging	Limited cell types The PDMS material of chip device may influence drug testing Low throughput for drug screening in the viral‐infected organ chips
Animal models	Widely used in evaluating preclinical therapeutic drugs or vaccines Studying organism responses to viral infection and pathogenesis Available for viral infection by gene editing (e.g., hACE2 transgenic mice) Nonhuman primates possess similar physiology and immunology to humans	Many animals are not the natural host for SARS‐CoV‐2 (e.g., rodents) Exhibiting varying susceptibility and different symptoms from human Limited throughput Difficult to real‐time imaging High financial costs and complex husbandry requirements in animal biosafety level 3 (BLS‐3) lab Ethical issues

## 5 Conclusions and Perspectives

SARS‐CoV‐2 has caused a severe worldwide outbreak and challenged humanity. The rapid spread and evolution of this pathogen and the increasing number of cases emphasize the urgent need to establish in vitro biomimetic human organ models to probe host‐virus interactions and to develop efficient therapeutics. Stem cell‐derived organoids and organs‐on‐chips have both shown the ability to recapitulate human pathophysiology and host responses to SARS‐CoV‐2 infection, helping in drug screening to identify promising COVID‐19 therapeutics. In particular, organoids provide the opportunity to study the tissue tropism of SARS‐CoV‐2 and can be used in high‐throughput drug screening. Moreover, organoids derived from different donors may lead to a better understanding of the virus behavior among humans. Organs‐on‐chips can recreate organ functional units and cell–cell interactions by integrating tissue–tissue interfaces, immune cells, and mechanical cues. They provide a visualization of how SARS‐CoV‐2 infects human cells and triggers the complex host‐virus interplay and the immune response. Compared with traditional cell cultures and animals models, these physiologically relevant 3D organ models provide a more elaborate translation to the human responses, including virus transmission and systemic pathology. These models have helped to combat COVID‐19 and may be used in future epidemics, ultimately expediting the development of vaccines and therapeutics.

### 5.1 Virus Transmission

The vast majority of virus transmission is horizontal between individuals within the at‐risk population and occurs through direct/indirect contact, droplets, common vehicle, or airborne transmission. It has been generally recognized that the dominant route of SARS‐CoV‐2 transmission is respiratory. Some studies have speculated that SARS‐CoV‐2 can be transmitted via the fecal‐oral route by COVID‐19 patients with gastrointestinal symptoms, but this has not been confirmed. Animal experiments have provided some evidence of SARS‐CoV‐2 infection in domestic pets and farm animals, such as cats, dogs, and ferrets.^[^ ^137^, ^138^, ^139^ ^]^ Several studies have suspected the animal‐to‐human transmission of SARS‐CoV‐2, for example the mink.^[^ ^140^, ^141^ ^]^ There have also been several clinical cases reported the early nasopharyngeal positivity of viral RNA after delivery in neonates, as well as placental infection by SARS‐CoV‐2, indicating the possibility of vertical transmission of the virus. As yet, the exact vertical or transplacental transmission of the virus or other transmission routes is unclear. Organ chips could offer a rapid and low‐cost platform for studying viral infection and transmission dynamics in a realistic manner. A human lung airway chip was used to model human‐to‐human transmission and evolution of influenza virus, which provide a useful tool for studying respiratory viral transmission and testing antiviral drug resistance.^[^ ^142^ ^]^ Using different modes of virus inoculation or diverse organ chips, such as the lung, gut, and placenta chips, the deep understanding of the pathogenesis and transmission of SARS‐CoV‐2 might be achieved. A chip model integrated with animal‐derived cells could also be used for studying cross‐species viral transmission. SARS‐CoV‐2 has rapidly evolved into several genetic variants, and some variants have increased transmissibility and a higher viral load in the human body, which posed an increased risk to global public health.^[^ ^6^, ^7^, ^8^ ^]^ The molecular basis for the rapid spread of SARS‐CoV‐2 and its escape from host immunity is largely unknown. Organ chips may provide a potential platform for the in‐depth and real‐time investigation of viral person‐to‐person transmission and evolution by passaging viruses from chip to chip, which could help to identify possible intervention strategies to cope with emerging variants.

### 5.2 Drug and Vaccine Development

Drugs and vaccines are effective ways to prevent the rapid spread of COVID‐19. Although some drugs and vaccines have been approved for human use, their efficiency has been limited owing to rapid virus evolution. The Delta variant has shown moderate vaccine resistance, thus there is an urgent need for the development of more effective drugs and vaccines to brace for this issue. Notably, many candidate drugs or vaccines that worked in animals often fail in humans due to the distinctive responses among species.^[^ ^143^, ^144^ ^]^ Organoids and organ chip models can provide new insights into host responses to infection and disease pathogenesis, which may serve as potential alternatives to animal models and traditional cell cultures for accelerating drug and vaccine development. Human tonsil tissue‐derived organoids were recently developed to evaluate adaptive immune responses to rabies vaccine and SARS‐CoV‐2 vaccine.^[^ ^145^ ^]^ Organoids derived from patients or normal iPSCs also have proven to be valuable for studying disease pathogenesis and drug action. They may play a key role in preclinical drug screening, vaccine testing, and personalized medicine. In addition, organs‐on‐chips enable the evaluation of host immune responses by integrating multiple cell interactions, thus candidate anti‐inflammatory drugs or inhibitors targeting host inflammation to viral infection could be tested. A human model of lymphoid follicle was established by self‐assembly of primary blood lymphocytes in 3D matrix using organ chips and used for preclinical vaccines evaluation.^[^ ^146^ ^]^ These human immune organoid or organ chip models provided useful tools for studying immunologic mechanism and rapidly testing candidate vaccines or drugs.

### 5.3 Systemic Pathology

COVID‐19 is a systemic disease involving SARS‐CoV‐2 infection and replication in multiple organs, such as the lung, liver, brain, heart, blood vessels, kidney, and intestine. The development of more complex systems is needed to explore the interactions among different organs and virus. Multi‐organs‐on‐a‐chip may offer a unique opportunity to recapitulate organ–organ crosstalk in an interconnected manner and model the systemic responses to infection, ultimately accelerating preclinical drug discovery and personalized therapies. Recently, the established linked alveolus‐BBB organ chip has provided a proof‐of‐concept to study SARS‐CoV‐2 infection in a multi‐organ context.^[^ ^125^ ^]^ In future, it may be amenable to integrated with other types of organ chip module for this systemic infectious disease research, which would help to deep‐understand the mechanisms of COVID‐19 and drug candidates screening.

**Figure 4:**
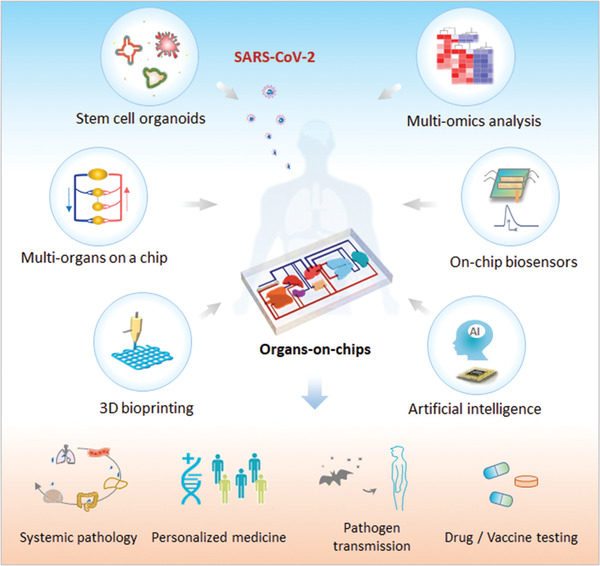
Schematic depiction of next‐generation human organ models to meet the needs of virology research. Organoids and organ chips are useful platforms for addressing COVID‐19 challenges, but they still have limited capacities. Advanced human organ models could be realized by integrating organoids and organ chips with other approaches, including 3D bioprinting, multi‐omics, biosensors, and artificial intelligence. It is expected the next generation of human organ models will uncover signatures in pathogen transmission and provide new opportunities for systemic response analysis, personalized medicine, and novel drug and vaccine development.

Organoids and organs‐on‐chips have displayed extensive utility in research on COVID‐19 and viral infections, but models incorporating various cell components such as the vascular and immune systems are still lacking. The alone model has a limited ability to meet the broad range of requirements needed to address emerging and re‐emerging pandemic diseases. The newly developing organoids‐on‐a‐chip technology has exhibited great potential in building higher fidelity organ models. In the future, this technology can be implemented with additional microfluidic elements, such as on‐line biosensors, 3D printing, gene editing, high‐content microscope images, and multi‐omics, to create more complicated and rapid fabrication of next‐generation organ models (**Figure** **4**). We envision that these advanced organ models might be capable of simulating a realistic host–pathogen interaction in vitro, thus facilitating the development of potential therapies to address the major challenges of COVID‐19 or the next pandemic.

## Conflict of Interest

The authors declare no conflict of interest.
